# Bread and Milk

**Published:** 1860-04

**Authors:** 


### HALL'S JOURNAL OF HEALTH.

**Our Legitimate Scope is almost boundless: for whatever begets pleasurable and harmless feelings, promotes Health; and whatever induces disagreeable sensations, engenders Disease.**

WE AIM TO SHOW HOW DISEASE MAY BE AVOIDED, AND THAT IT IS BEST, WHEN SICKNESS COMES, TO TAKE NO MEDICINE WITHOUT CONSULTING A PHYSICIAN.

**Vol. VII.] APRIL, 1860. [No. 4.**

### BREAD AND MILK.

THE "staff of life" is different in different countries. In Kentucky, it is "hog and hominy," and that it "agrees" pretty well with the people, is evidenced from the fact, that in our native county, and which may be considered the model county of the State, in respect to the richness of its soil, the weight of its pigs, the fatness of its cattle, and the unsurpassable beauty of its woodland, blue-grass pastures, dotted with thousands and tens of thousands of cattle grazing, in the brightness of a June morning—in this same county of Bourbon, whence comes the best whisky, the best bacon, and the best beef in the land, there are also found the best specimens of giant growth among its men, not only in body, but in mind. As to physical development, there are whole families whose average hight is six and a half feet. As to mind, let us see, taking Paris as a center, with a "spoke" (of a wheel—a good many may not know what a radius is) twenty miles long, there have grown up into life, notice and renown, such men as, putting the Editor of the JOURNAL OF HEALTH first for the convenience of a gradual accretion of greatness, "topping off" with the greatest man of all nations of the present age; and lest it might be forgotten, we will repeat; there is, in the first place, the Editor of HALL'S JOURNAL OF HEALTH to begin with; next, Tom Corwin, the wagon-boy; Dr. Durbin, the great Methodist divine, and who now maintains the same relative position here in New-York; and Dr. T. A. Mills, the right-hand man as to mind and efficiency in the New School Presbyterian Church, pronounced its "leader" by the New-York *Observer* and the *Evangelist* also. Then there is Dr. B. W. Dudley the elder, who, as a successful surgeon and physician, has had no equal in modern times, his great agents being nature, brown bread, and warm water; he having been our medical teacher, we took our cue from him, hence our large quantum of common-sense—theoretically, for does any body suppose we practice half what we preach in this JOURNAL? very clear of it. Why should we? We have good health, and always have had it, and why should we be bothered with innumerable "rules and regulations," making of ourselves a very slave? It is one of the very worst kind of tyrannies to bring yourself into the predicament of breathing and talking by rule. With a few cardinal principles, a man, as far as the preservation of health is concerned, may live above rules, at least until he gets old and rickety; then, indeed, he must be propped up and live by "rote;" indeed, the old and infirm will always live the longer by so doing.

One of Dr. Dudley's favorite counsels was:

"Young gentlemen, observe Nature and obey her laws; let her alone; never interfere with her, but follow out her indications."

Those few plain words embrace the whole range of practical medicine; they include anatomy, physiology, melancholy, obstetrics, botany, and medicamentum!

"OBSERVE NATURE AND FOLLOW HER INDICATIONS!"

These half-a-dozen words are the foundation of all practical hygiene; and if the young gentlemen from our medical schools could retain their perfection in anatomy and physiology, forgetting every thing else, and would observe Nature accurately and follow wisely her indications, nine tenths of the apothecaries of the nation would have to close their doors within a year, especially if the people had the minutest mite of common-sense, and would show it, by never taking even a homeopathic pill without special medical advice.

It is interference with nature which kills multitudes of those who die of disease, as it is the defiance of her laws which made those multitudes sick.

As we were saying, making Paris, Bourbon county, Kentucky a center, with a radius of twenty miles, a circumference will be described, within which have sprung into honorable notoriety, a young multitude (or baker's dozen) of names, (one of which is ours, let it be remembered,) of which any age or nation might be proud. Some of these we have mentioned; among the others worthy, are Alexander, the Hawaiian Missionary, and "Bob Breckenridge," the John Knox of modern Presbyterianism, the Politico-Divine, whose great mind and greater heart have lately so grandly commended themselves to the sober, conservative, and patriotic masses of the nation by his Union letter to his nephew, the Vice-President of the United States, which letter can well be placed side by side with an ecclesiastical document of his composing, the (Presbyterian) famous "Act and Testimony," the adoption and adherence to which, quieted the troubled waters of his Church, and at the same time laid the foundation for that peace and prosperity which now crowns Old School Presbyterianism, and which aids in making it one of the most conservative of all Christian sects; conservative in principle, in practice, and in wide-reaching influences. Why, the Union can't dissolve with such a leaven within it? Well, we have stuck to the text with a vengeance.

If any body can tell what connection there is between bread and milk and "Bob Breckenridge," it is more than we can do, unless it be the connection of disconnection, a kind of antipodal Union; for bread and milk suggests milk and water, which in turn is associated with that multitude which no man can number, of dodling wishy-washy nonentities who are of no account to themselves or any body else, the dead-weights to all that is great and good. Well, the "connection" between "Bob Breckenridge" and milk and water is impossible, and we rather think this is a good place to break off at, and we will toddle back to first principles, at least so far as to say, that within the famed circumference, not that magnificent unique square which the New-York *Times* described during the late war, other names still have become famous, such as Drs. Charles Caldwell and John Esten Cook, of Transylvania University, Dr. Rice of Chicago, and last of all, the friend and neighbor and peer of Breckenridge, the friend of "The Constitution, the Union, and the enforcement of the Laws," and the friend of man—Henry Clay!

Perhaps, not until their greatness was achieved, did either of these names ever pass a day in Kentucky, in which they did not eat hog or hominy, or both, with perhaps a reasonable proportion of the spirit of "old Bourbon" besides; for when we were growing up with the great men named, it was considered a breach of politeness, of which the veriest boor would have been ashamed, not to place a bottle of whisky and a glass to every caller. How we all lived through it, may admit of debate; and as argument (domestic ones especially) is our mortal antipathy, by reason of the fact of our never failing to come out of it second-best, we leave it to our readers to debate at their leisure, and decide for themselves whether all of us survived by reason of the whisky antagonizing the destructive nature (so claimed by some) of the pork, or whether the pork was an antidote to the whisky, or whether, indeed, eating pork and drinking whisky daily, made a grand system of feeding, which gave splendid bodies and more magnificent minds. We ourselves ate the pork, but never "took to whisky-drinking," hence, possibly, the reason that the mind is so far ahead of the body.

The staff of life for New-England, the three articles daily used by the men who grew up over thirty years ago, were pork, molasses, and rum; but as the last two articles were the products of slave labor, which was against their principles, yet patronizing slave labor contrary to their principles, conscience, being systematically and deliberately violated, worried the mind. Now, no person can thrive or grow fat when the mind is distressed; it may be argued that this is the reason why there are so many thin, skinny Yankees scattered around every where. Not only lean in body but lean in morals and faith. At all events, they do say, that New-England is becoming more reprobate, going further away from the faith of their fathers, trending towards infidelity, and as Dr. Scudder says, a veritable pantheism; the Sabbath day worship of some of their theological lights being conducted without prayer or praise or reading of the "word," but a simple cold "speak" or address. Some may say that there is no connection between a dwarfed theology and a feeding on pork, rum, and molasses against the conscience. At the same time, it is an opinion gaining prevalence with thoughtful minds, that something is at work in eating out the stern piety and the Bible theology of glorious New-England of the olden time. While the land of the Puritans, with its codfish and potatoes, its Jamaica rum and pumpkin-pies, gives such men as Parker and Emerson and Phillips and Holmes and Garrison, Tennessee with its corn-bread and bacon, its potatoes and its beef, gives a Blackburn, a Gallaher, a David Nelson and a host of other worthies, with a Sam Houston and an Andrew Jackson to lead them.

Beef is the staff of life to sturdy John Bull, or rather beef and beer, and no such nation ever existed as that same grand English people! great for ages past and will be great for ages to come!

In China and Japan, rice is the alpha and omega of teeming millions, as bread and wine is that of the grand nation which has given to the world two Napoleons and a list of savans who shine in the coronet of France as stars of the first magnitude glitter in the clear sky of night.

But bread and milk is the staff of life to all juvenility; children thrive upon it the world over, until they reach a certain age, when more substantial food is needed, at least north of the tropics.

In Germany, sour krout (vinegar and cabbage) is the staff of life; and at once the image of a fat Dutchman rises before us with his smoke-pipe and his pot of Lager, all jolly, rubicund, and a mile round; but in a trice he vanishes from sight, in transcendental mazes lost!

But here come the Jews, whose staff of life is *not* pork, and a long line of patriarchs, and apostles, and prophets file away before us in holy memories; and coming down to later times we find them kings in music and in song, in painting and in finance, and the autocrat and the emperor besiege their doors, and say to a Rothschild, We can not go to war unless you let us have the money.

A long circumbendibus have we made; we have doubled and trebled the famous barn of Robin Hood, in order to come to a practical focus, and which common-sense might have taught any man with two ideas; it is this, national greatness and individual eminence do not depend materially on what a man feeds. Nor does robust health belong to a man mainly because he eats this and refuses that; but a wise providence has so ordered it, that the people of a clime may live and thrive and be happy and great by the products of that clime, and that in individual constitutions, there is an adaptability by which men can live in health any where, whether to the manor born or not, only if they eat and drink in moderation any of the good things about them. In short, the world was made for man, by that Father whose boundless goodness has provided for him all things richly to enjoy. Those who do not indulge in the wholesome variety which has been prepared for them, but insist on discarding this, that, and the other, finally come down in their vagaries, until a man writes a book of two hundred pages to prove that meats are forbidden, and that if we don't quit eating so much wheat-bread, the bone will become so brittle, that every time a man stumps his toe, said bones will snap like pipe-stems, closing by suggesting as the surest means of living long in vigorous health, that men should live almost exclusively on grapes. We know he made one convert, for sometime after, we chanced to drop in at the old Clinton Hall, where a phrenological lecture was grinding out. A black-headed, pale-faced, lantern-jawed young clerk was on the stand and the phrenologist's fingers were on his head. Silence prevailed! At length Sir Oracle spoke: "You are deficient in vitality and in brains, and the only safe plan for you to pursue is to take out-door exercise and live on grapes."

It is a notable coïncidence that these exclusives, either have no brains to begin with, or they grow weak in the upper story with marvelous rapidity on their watery diet. We can tell one of them on Broadway as far as we can see him, if a "him" at all; the sure signs are the parted hair, sprangling over a greasy coat-collar, with a dusty musty beard, a mile long, more or less, and that peculiar impudent look which is known at first sight; but if it be a "her," she is rendered distingué by the short hair, the short dress, the man's hat, and a man's brass.

It is a maxim of ancient date, misery loves company, and just as true, that kind loves kind, and birds of a feather flock together. The timbers of a ship dashed to pieces on the rocks, come together at last on the shore; it is on this principle perhaps, that wooden-headed people are found all in heaps, are vastly gregarious. Well, a wooden head and a weak head being pretty much the same thing, may account for the fact, that upon the announcement of any novelty to which the public are invited to attend, such as a Bloomer lecture, a woman's rights address, a phrenological examination, a health convention, or any thing of that nature, the assembly is sure to be made up of a "so-so" looking crowd; neither high nor low, but a kind of mediocrity, with the vulgar look or twang predominating; and if any one characteristic is in the ascendant, it is a kind of impudent swagger in the men; and in the women—"schuze me," as our little Alice says; he who speaks disrespectfully of a woman has no music in his soul, and is fit for treason, strategems, and spoils; therefore we won't do it, especially as the most tantalizing sight we ever had, was a jaunty Bloomer of seventeen, black eyes, and a world of curls.

But how is it that when we meet a vegetarian, he is almost sure to be a phrenologist, a free lover, a root-doctor, a woman's rights, a mesmerist, a spiritualist, a socialist, a cold waterist, a ranting abolitionist, an abnegator of the Bible, the Sabbath-day, and "the religion of his fathers!" The Editor is persuaded that observation will carry him out in the assertion, that in the vast majority of cases, a man who advocates one of these isms, will, if pressed, advocate them all. We say this in no spirit of ridicule or intolerance, but utter it as a fact, with a view to drawing a wholesome, truthful, and practical lesson therefrom; and it is this, that in health, in dietetics, in ethics, in politics and religion, extreme views are always unsafe, disorganizing, and destructive; and that the wisest plan, especially for the young, for women, and for all uncultivated minds is to make the fact that a thing is radical, extreme, or new, a most conclusive reason for keeping aloof from it, until the clergyman or other educated or mature-minded person of the place, has given it a thoughtful examination and an unequivocal approval.

